# lncRNAs PVT1 and HAR1A are prognosis biomarkers and indicate therapy outcome for diffuse glioma patients

**DOI:** 10.18632/oncotarget.20226

**Published:** 2017-08-12

**Authors:** Hecun Zou, Lan-Xiang Wu, Yonglong Yang, Shuang Li, Ying Mei, Yong-Bin Liu, Lihua Zhang, Yu Cheng, Hong-Hao Zhou

**Affiliations:** ^1^ Institute of Life Sciences, Chongqing Medical University, Chongqing 400016, China; ^2^ Department of Clinical Pharmacology, Xiangya Hospital, Central South University, Changsha 410008, China; ^3^ Haikou People’s Hospital and Affiliated Haikou Hospital of Xiangya Medical School, Central South University, Haikou 570311, China; ^4^ Department of Neurosurgery, Xiangya Hospital, Central South University, Changsha 410008, China

**Keywords:** PVT1, HAR1A, prognosis biomarker, therapy outcome, diffuse glioma

## Abstract

Diffuse gliomas are well known malignant brain tumors. Long non-coding RNAs (lncRNAs), a type of RNA transcript with more than 200 nucleotides, involve in tumorigenesis and development of various cancers. This study focused on identifying differentially expressed lncRNAs in gliomas based on gene expression profiling, and chose certain lncRNAs PVT1, CYTOR, HAR1A and MIAT, which changed with significant differences. Further analysis of TCGA and GEO data revealed that the expressions of PVT1 and CYTOR were up-regulated, while HAR1A and MIAT expressions were down-regulated in gliomas. Their expression patterns were validated in an independent cohort containing 98 glioma specimens and 12 non-tumor tissue controls. High expression of PVT1 and CYTOR as well as low HAR1A and MIAT expression were associated with high Ki-67 level and more *TP53* mutation. Kaplan-Meier survival curve and Cox regression analyses showed that glioma patients with high PVT1 expression or low HAR1A expression had poor survival outcome, aberrantly expressed PVT1 and HAR1A could be the independent prognosis biomarkers for glioma patients. Moreover, down-regulation of PVT1 and up-regulation of HAR1A contributed to improve the survival of patients who received chemotherapy and radiotherapy. These results implied that these four lncRNAs might play important role in diffuse gliomas progression, particularly, PVT1 and HAR1A could be explored as promising biomarkers for diagnosis, prognosis and target therapy of diffuse gliomas.

## INTRODUCTION

Diffuse gliomas (including astrocytoma, oligo-dendroglioma, oligoastrocytoma, and glioblastoma), are the most common types of primary brain and central nervous system (CNS) tumors. According to cancer statistics, the mortality due to diffuse gliomas is the highest among brain and CNS tumors, the median survival duration of glioblastoma (GBM) patients is less than 15 months, despite the comprehensive treatment combining surgical resection and adjuvant chemoradiotherapy [1]. The pathogenesis of gliomas are very complicated, involving aberrant activation of proto-oncogenes and inactivation of tumor suppressors [2]. The 2016 WHO classification of CNS tumors (2016 CNS WHO) first uses molecular characteristics plus histology to define many tumor entities, such as *IDH* mutation and *EGFR* amplification, formulating a concept of how tumor diagnosis should be constructed in the molecular era [3]. Thus, it is urgent to look for novel biomarkers for diagnosis and prognosis of diffuse gliomas.

Long non-coding RNAs (lncRNAs) are commonly defined as a type of non-protein coding RNA transcript with more than 200 nucleotides. Several lncRNAs have been identified to play crucial roles in modulating relevant gene expression through transcriptional or post-transcriptional regulation [4]. Dysregulation of lncRNAs has been linked to different diseases including cancer. Emerging evidences have revealed that lncRNAs could function as proto-oncogenes or tumor suppressors, which involved in tumorigenesis and development of various cancers, including diffuse gliomas [5]. Particularly, the cancer specific lncRNA profiles have been reported, and lncRNAs can be detected by quantitative real-time PCR (qRT-PCR) method in a relatively stable form. In view of their high specificity and stability, several lncRNAs are thought to be potential biomarkers in cancers [6]. Additionally, certain lncRNAs are of diagnostic and prognostic significance, indicating a promising role in the prognosis and target therapy of cancer [7]. Therefore, understanding the expression pattern and biological function of lncRNAs in gliomas may enable us to find a new therapeutic strategy for diffuse glioma patients.

In the present study, we aimed to identify differentially expressed lncRNAs based on gene expression profiling, and selected certain lncRNAs with significantly differential expression in diffuse gliomas. The correlations of these lncRNAs’ expression with clinical pathological characteristics of glioma patients, including chemoradiotherapy outcome, have been studied in order to reveal the underlying clinical significance.

## RESULTS

### Differentially expressed lncRNAs between glioblastomas and non-tumor controls

**Figure 1:**
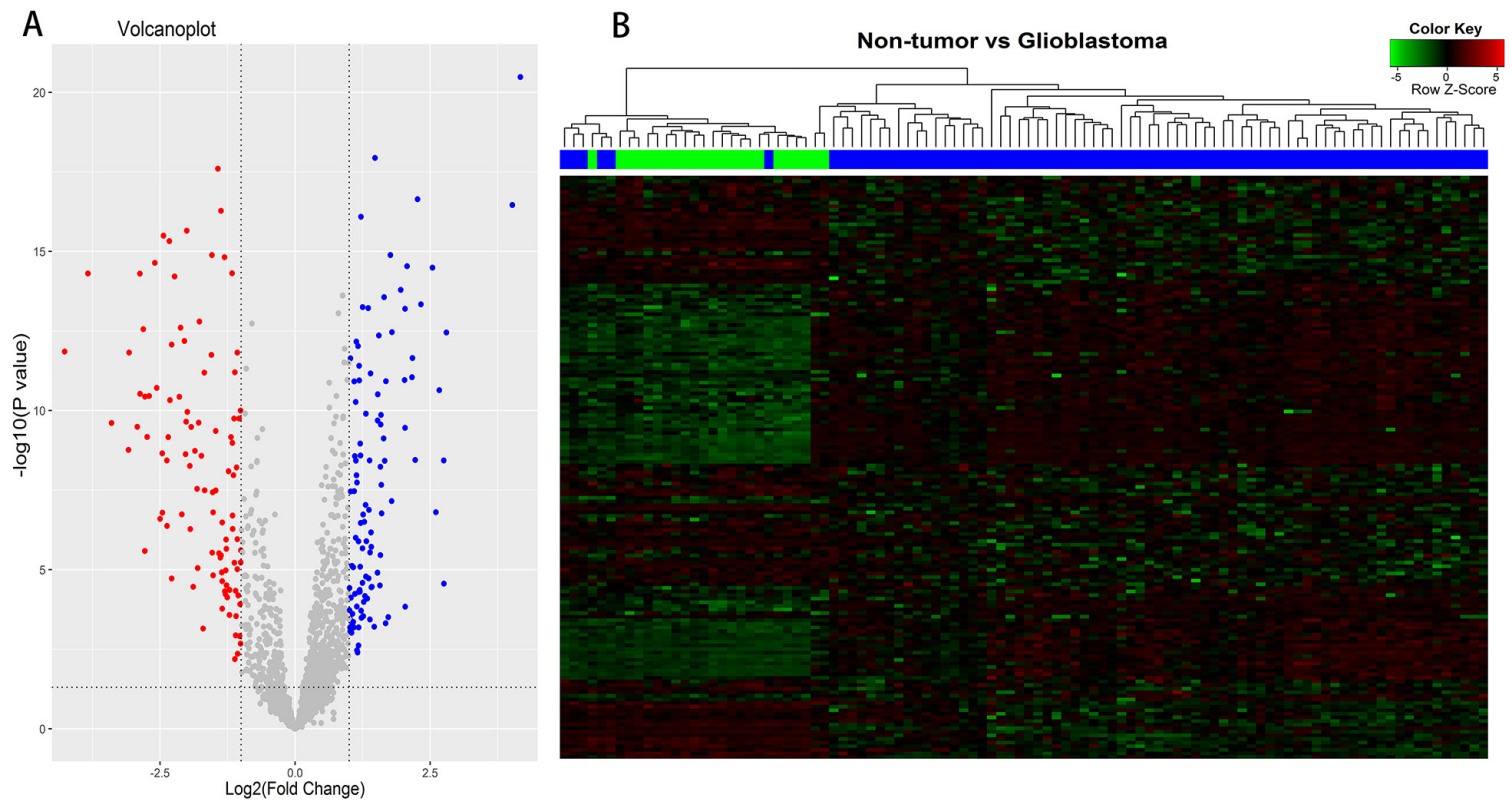
Differentially expressed lncRNAs (DELs) analysis between glioblastomas and non-tumor controls **(A)** Volcano plots of the DELs, consisting of 81 up-regulated and 82 down-regulated lncRNAs. **(B)** Clustering and heat-map of DELs, up-regulated lncRNAs are clustered in the magenta-shaded areas, and the green-shaded areas indicate down-regulated lncRNAs. Dendrogram reflect relationship among samples; the blue bars consist of glioblastoma samples and green bars indicate non-tumor controls.

According to Affymetrix annotations of probe sets and the Ref-Seq annotations of lncRNAs, we screened out 1950 probe sets (corresponding to 1303 lncRNA genes) within Affymetrix HG-U133 Plus 2.0 arrays, after excluding pseudogenes (Supplementary Table 1). Application of bioinformatics analysis, we investigated differentially expressed lncRNAs (DELs) between 77 glioblastoma samples and 23 non-tumor controls of the GEO dataset (GSE4290). Based on the cut-off point (|logFC| >1; *P* <0.05), a total of 163 DELs were identified, of which 81 were up-regulated and 82 were down-regulated (Figure 1A). Among the differentially up-regulated lncRNAs, we selected PVT1 (Pvt1 oncogene) and CYTOR (cytoskeleton regulator RNA) (logFC =1.395, *P* =6.82E-12 for PVT1; logFC =1.246, *P* =2.14E-06 for CYTOR), which were reported previously [8, 9]. Among the down-regulated lncRNAs, HAR1A (highly accelerated region 1A) and MIAT (myocardial infarction associated transcript) were presented with high value of an absolute log2(fold-change) (logFC =-2.873, *P* =2.98E-11 for HAR1A; logFC =-2.459, *P* =1.61E-07 for MIAT). Thus, we chose these lncRNAs PVT1, CYTOR, HAR1A and MIAT for further study. Hierarchical Cluster analysis was performed according to the expression values of differentially expressed lncRNAs, and heat-map was exhibited in Figure 1B. It showed differential lncRNAs expression modes between glioblastoma samples and non-tumor controls, indicated that noticeable differences existed between two groups.

### The expression of PVT1, CYTOR, HAR1A and MIAT in glioma datasets

**Figure 2:**
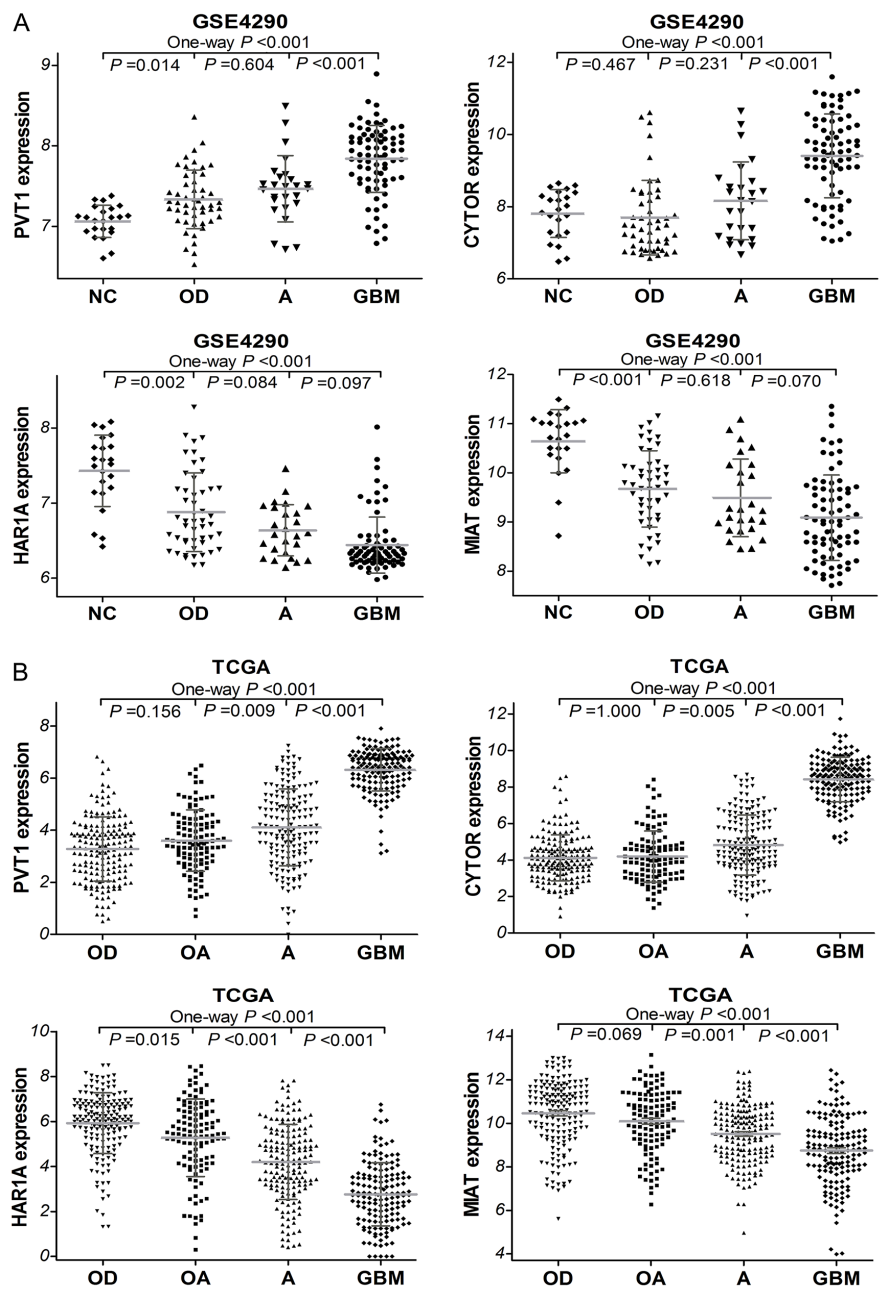
The expression of PVT1, CYTOR, HAR1A and MIAT in glioma samples of GSE4290 **(A)** dataset (50 of OD, 26 of A, 77 of GBM, and 23 of NC) and TCGA **(B)** dataset (175 of OD, 115 of OA, 170 of A, and 152 of GBM). OD, oligodendroglioma; OA, oligoastrocytoma; A, astrocytoma, GBM, glioblastoma.

To investigate the expression levels of these four lncRNAs in diffuse gliomas, we first analyzed glioma gene expression dataset (GSE4290). As shown in Figure 2A, PVT1 and CYTOR expressions were remarkably increased in glioma samples compared with non-tumor controls (both *P* <0.001). GBM demonstrated a significant higher CYTOR and PVT1 expression levels than astrocytoma (A, *P* <0.001), and oligodendroglioma (OD, *P* <0.001). The expressions of HAR1A and MIAT were significantly decreased in glioma samples compared to non-tumor controls (both *P* <0.001). Moreover, the independent glioma RNAseq data from TCGA was employed to perform further analysis, and we found that the expressions of PVT1 and CYTOR were noticeably higher in GBM samples than in lower grade gliomas (LGGs, all *P* <0.001; Figure 2B) that refer to some diffuse glioma subtypes including astrocytoma, oligodendroglioma and oligoastrocytoma (OA). HAR1A and MIAT expression were significantly lower in GBM samples than in LGGs (all *P* <0.001). However, no significant difference of these four lncRNAs expression was observed among oligodendroglioma, oligoastrocytoma or astrocytoma, which were consistent with the result from GSE4290 dataset. These findings indicated that CYTOR, PVT1, HAR1A and MIAT were aberrantly regulated and might play an important role in diffuse gliomas progression.

**Figure 3:**
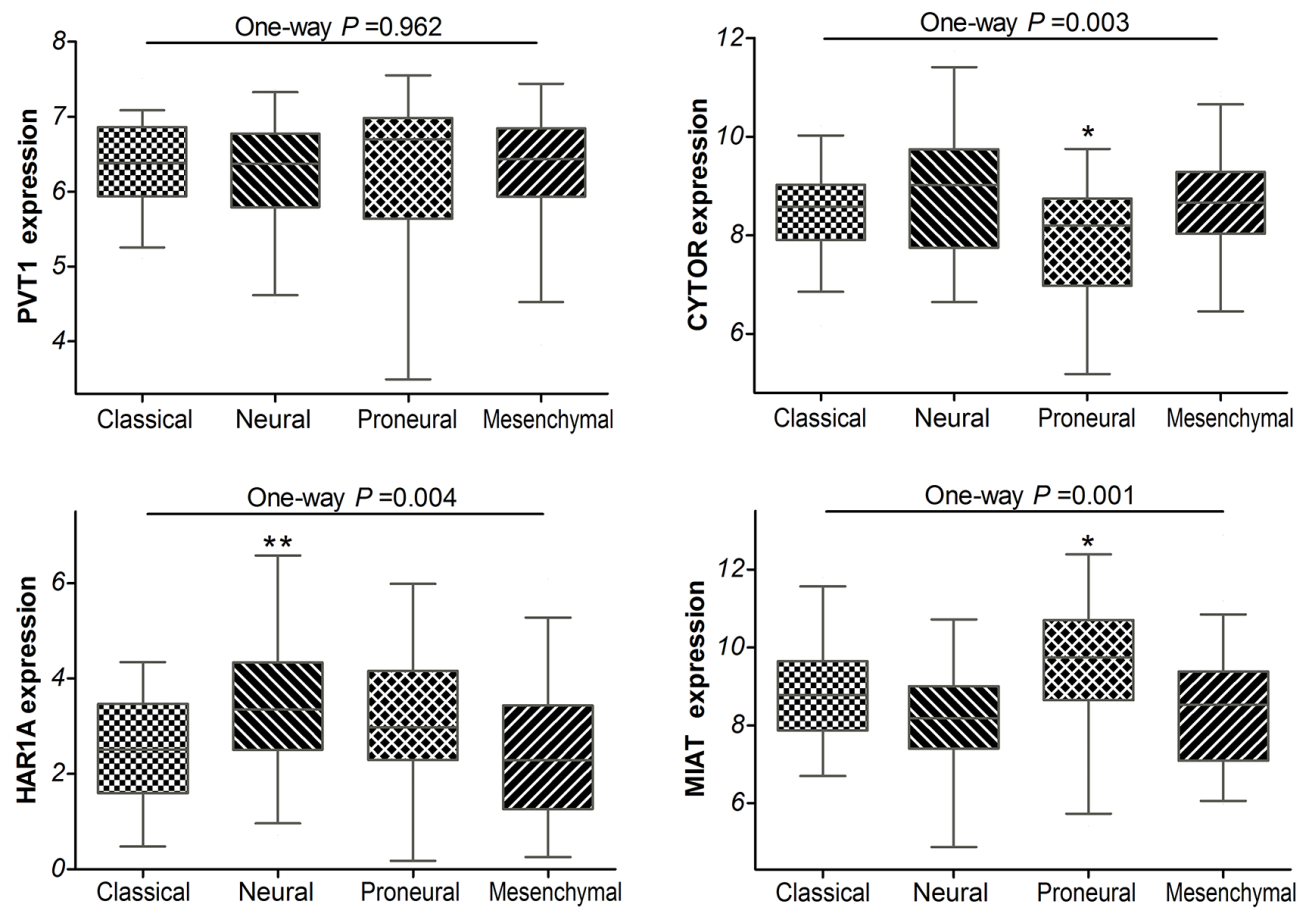
Box-plot of the PVT1, CYTOR, HAR1A and MIAT expressions in the classical, neural, proneural and mesenchymal subtypes of glioblastoma samples within TCGA * means P<0.05, ** means P<0.01.

Additionally, the TCGA network have defined classical, neural, proneural, and mesenchymal subtypes by gene expression–based molecular stratification of GBM [10]. We applied the classification criterion to describe the expression of PVT1, CYTOR, HAR1A and MIAT in glioblastoma subtypes. The analyses showed that there were significant differences in CYTOR, HAR1A and MIAT expression levels among the four subtypes of GBM in TCGA (*P* =0.003, *P* =0.004 and *P* =0.001, respectively; Figure 3). Especially, as compared with classical subtypes, CYTOR was less expressed in the proneural subtypes (*P* =0.017), while MIAT was preferentially expressed in the proneural subtypes (*P* =0.029); HAR1A was preferentially expressed in the neural subtype (*P* =0.005). By definition, the proneural subtype is associated with higher amplification of *PDGFRA* and more *IDH1* mutations; neural subtype is closely related with the expression of neuron markers such as NEFL, GABRA1, SYT1 and SLC12A5 [10, 11]. The results implied that CYTOR and MIAT may be associated with amplification of *PDGFRA* and *IDH1* mutations, HAR1A may be related with neuron markers in glioblastomas.

### Aberrantly expressed PVT1 and HAR1A are associated with poor survival of glioma patients

**Figure 4:**
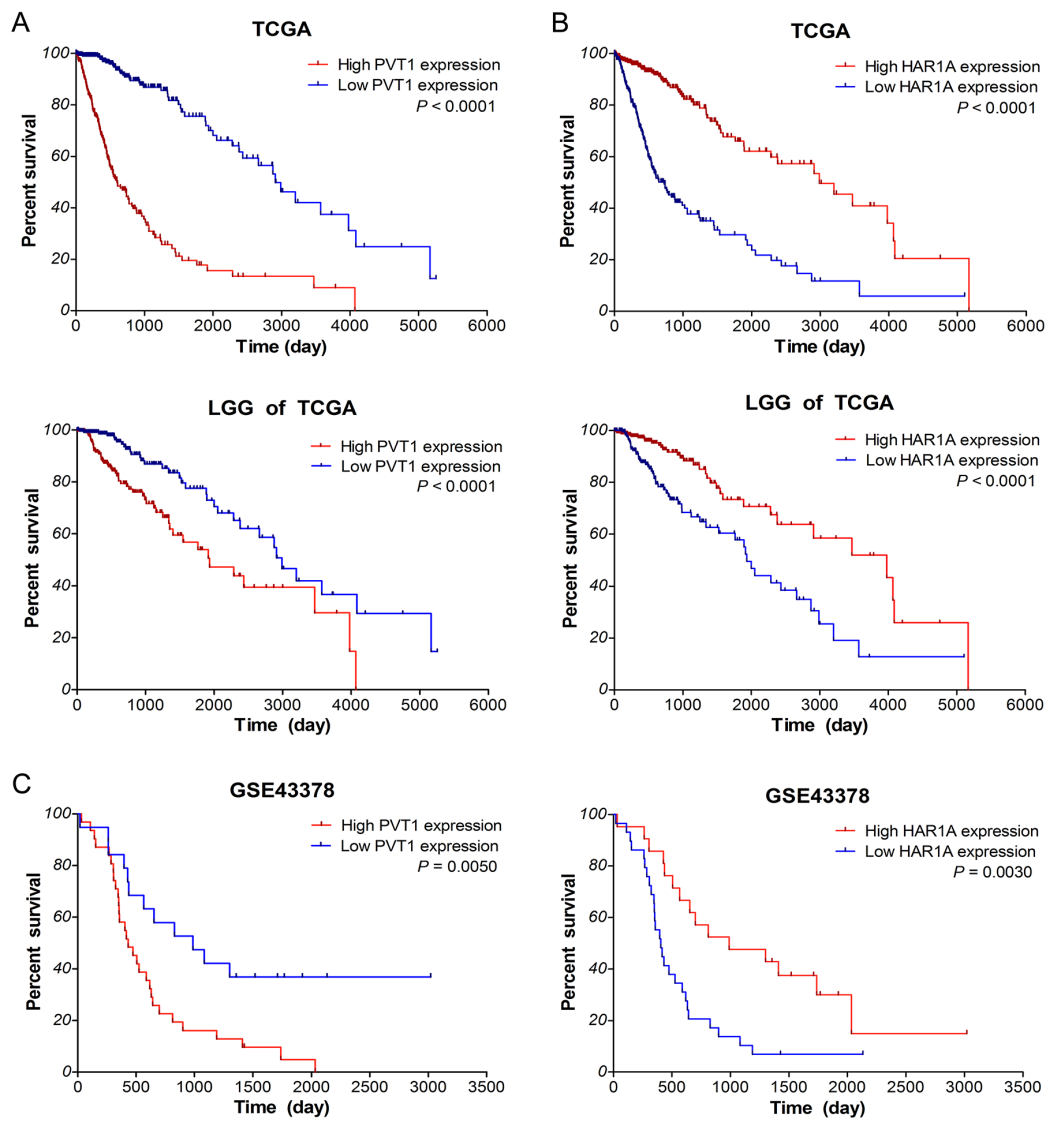
Up-regulated PVT1 and down-regulated HAR1A correlate with poor overall survival of glioma patients Kaplan–Meier survival curve analyses with a log-rank comparison were performed based on PVT1 **(A)** and HAR1A **(B)** expression in gliomas samples of TCGA along with LGG subtypes, and gliomas samples of GSE43378 **(C)** datasets.

Next, we investigated the correlation between lncRNA expression and overall survival (OS) of glioma patients by Kaplan–Meier survival analysis with a log-rank comparison in the independent glioma gene expression data of TCGA and GEO datasets. According to the mean value of gene expression, glioma patients were divided into two groups: low expression group and high expression group. As shown in Figure 4A-4B, within glioma samples of TCGA along with LGG subtypes, the overall survival of glioma patients with high PVT1 expression was remarkably worse than that of the low expression patients (both *P* <0.0001). Survival of glioma patients with low HAR1A expression was significantly worse than that with high expression (both *P* <0.0001). However, as for lncRNA CYTOR and MIAT, there was no significant association between their expression and the survival of glioma samples in TCGA (*P* >0.05, Supplementary Figure 1). Meanwhile, similar results were obtained from the GSE43378 dataset (*P* =0.0050 for PVT1, *P* =0.0030 for HAR1A, Figure 4C). All together, these results suggested that high PVT1 expression and low HAR1A expression were associated with poor survival outcome of glioma patients.

Furthermore, univariate Cox regression analysis of overall survival of glioma samples within TCGA showed that high PVT1 expression (*P* <0.001), low HAR1A expression (*P* <0.001), increased age (both *P* <0.001), high karnofsky performance score (KPS; both *P* <0.001) and WHO grade (all *P* <0.001 for II/IV and III/IV), and advanced histological types (all *P* <0.001 for OD/GBM, OA/GBM and A/GBM) were the risk factors associated with prognosis (Table 1). Subsequent multivariate analysis results revealed that high PVT1 expression (HR: 2.539, *P* <0.001) and low HAR1A expression (HR: 1/0.625=1.6, *P* = 0.021) were the independent prognosis factors for survival of glioma patients, in addition to increased age, high KPS and grade. Similar results were also obtained from the Cox regression analysis within GSE43378 dataset (data not shown). These data indicated that up-regulated PVT1 and down-regulated HAR1A could be the independent prognosis biomarkers for diffuse gliomas.

**Table 1:** Cox regression analysis of PVT1 and HAR1A in glioma samples of TCGA dataset

TCGA	PVT1				HAR1A			
Variables	Univariate model		Multivariate model		Univariate model		Multivariate model	
	HR	*P* value	HR	*P* value	HR	*P* value	HR	*P* value
**High expression**	6.386	<0.001	2.539	<0.001	0.246	<0.001	0.625	0.021
**Gender, F / M**		0.791				0.791		
**Age at diagnosis**	1.077	<0.001	1.032	<0.001	1.077	<0.001	1.033	<0.001
**KPS score**	0.941	<0.001	0.966	<0.001	0.941	<0.001	0.969	<0.001
**Grade II/IV**	0.051	<0.001	0.246	<0.001	0.051	<0.001	0.147	<0.001
**Grade III/IV**	0.147	<0.001	0.485	<0.001	0.147	<0.001	0.406	<0.001
**Histological types**								
**OD / GBM**	0.073	<0.001		>0.050	0.073	<0.001		>0.050
**OA / GBM**	0.085	<0.001		>0.050	0.085	<0.001		>0.050
**A / GBM**	0.140	<0.001		>0.050	0.140	<0.001		>0.050

HR, hazard ratio; F, female; M, male; KPS, karnofsky performance score; OD, oligodendroglioma; OA, oligoastrocytoma; A, astrocytoma.

### Validations of the PVT1, CYTOR, HAR1A and MIAT expressions in diffuse glioma specimens

**Figure 5:**
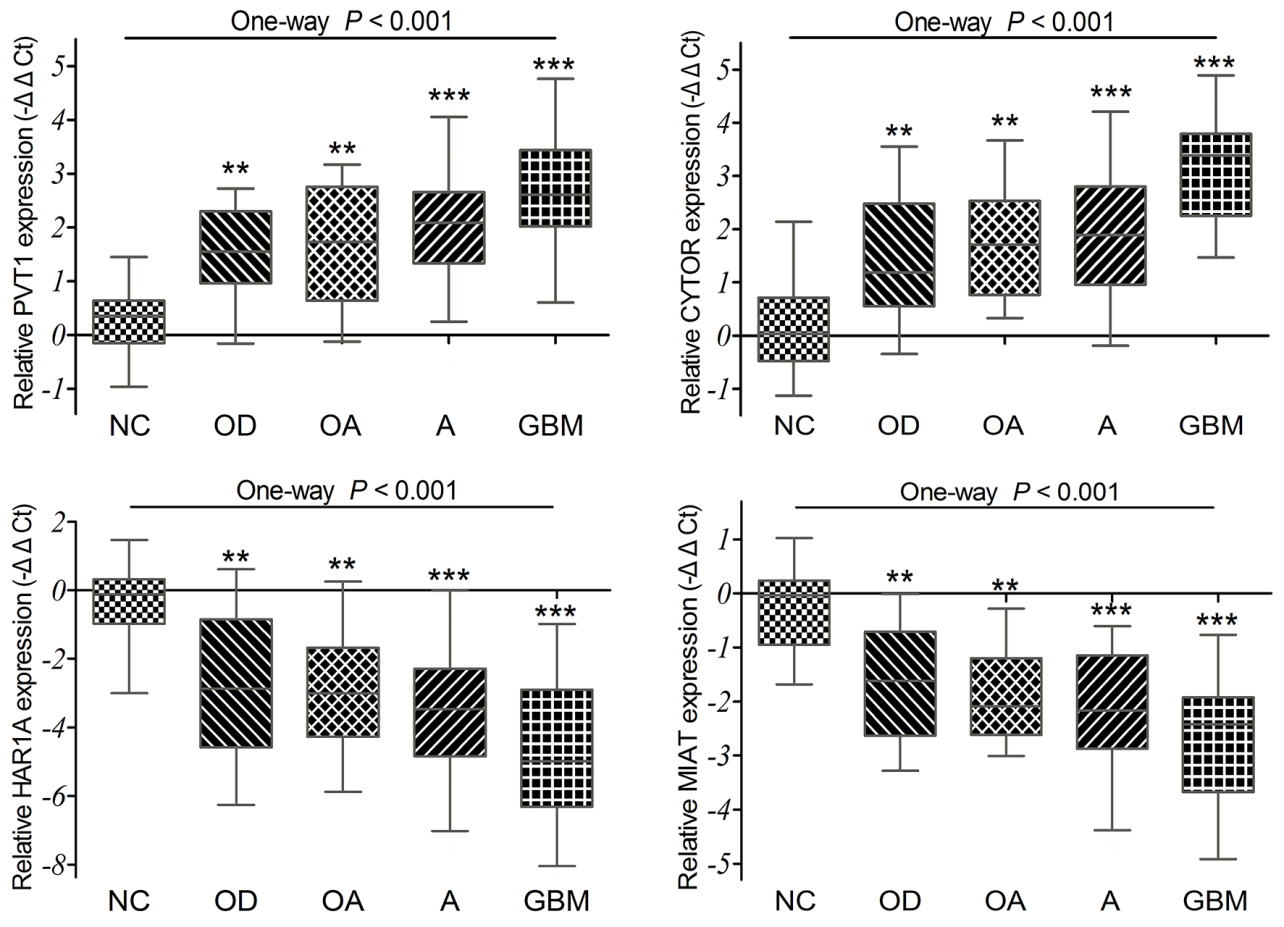
The qRT-PCR analysis of relative PVT1, CYTOR, HAR1A and MIAT expressions in 98 diffuse glioma specimens and 12 non-tumor brain tissues (NC) *P* =0.002 for PVT1, *P* <0.001 for CYTOR, *P* =0.002 for HAR1A, *P* =0.008 for MIAT between OD and GBM. *P* =0.015 for PVT1, *P* =0.002 for CYTOR, *P* =0.016 for HAR1A, and *P* =0.044 for MIAT between OA and GBM. *P* =0.011 for PVT1, *P* <0.001 for CYTOR, *P* =0.009 for HAR1A, and *P* =0.054 for MIAT between A and GBM.

To confirm the differential expression of four lncRNAs exactly, we detected their expression levels in 98 cases of diffuse glioma specimens and 12 cases of non-tumor brain tissues by qRT-PCR. Our results showed that the relative expression levels of CYTOR and PVT1 were up-regulated, while HAR1A and MIAT were significantly down-regulated in diffuse glioma specimens compared to non-tumor tissues (all *P* <0.001; Figure 5). GBM displayed remarkably higher PVT1 and CYTOR expression as compared with oligodendroglioma (*P* =0.002 and *P* <0.001), oligoastrocytoma (*P* =0.015 and *P* =0.002), and astrocytoma (*P* =0.011 and *P* <0.001). GBM also displayed statistically lower HAR1A and MIAT expression as compared with oligodendroglioma (*P* =0.002 and *P* =0.008), oligoastrocytoma (*P* =0.016 and *P* =0.044), and astrocytoma (*P* =0.009 and *P* =0.054). But no significant difference was observed among oligodendroglioma, oligoastrocytoma and astrocytoma (all *P* >0.05). Together with above analyses of TCGA and GEO datasets, all these data indicated that PVT1 and CYTOR were up-regulated, HAR1A and MIAT were down-regulated in diffuse gliomas.

### Correlations of the lncRNAs expression with pathology characteristics of glioma patients

On the basis of 2016 CNS WHO classification, the primary clinical and molecular pathology characteristics of diffuse glioma patients were listed in Supplementary Table 2. The mean value of relative lncRNAs expression was used as cut-off point, glioma patients were divided into low expression group and high expression group. As shown in Table 2A-2B, high expressions of PVT1 and CYTOR were statistically correlated with high Ki-67 in diffuse glioma specimens (*P* =0.002 and *P* =0.014). Low HAR1A and MIAT expressions were significantly correlated with high Ki-67 (*P* =0.008 and *P* =0.001). More p53 (indicate *TP53* mutation) was a relative risk factor for diffuse glioma patients with low expression of HAR1A (*P* =0.001) and MIAT (*P* =0.001). High PVT1 and CYTOR expressions were statistically correlated with more p53 in diffuse glioma specimens (*P* =0.003 and *P* =0.007). Meanwhile, high CYTOR expression (*P* =0.005) and low HAR1A expression (*P* =0.032) were correlated with *IDH* mutation in diffuse glioma specimens. However, no significant relationship was found between the lncRNAs expression and other pathology parameters including age, gender, KPS score, GFAP, *MGMT* promoter methylation, and 1p/19q codeletion (all *P* >0.05). As might be expected, Ki-67 is a nuclear protein that may be necessary for cellular proliferation [12]. *TP53* gene encodes tumor suppressor protein p53, which responds to diverse cellular stresses to regulate expression of multiple target genes. Mutations in *TP53* gene are associated with a variety of human cancers including gliomas [13]. These findings implied that up-regulated PVT1 and CYTOR, along with down-regulated HAR1A and MIAT might play an important role in tumor progression of diffuse gliomas.

**Table 2A:** Correlations between lncRNAs (PVT1 and CYTOR) expression and pathology parameters of diffuse gliomas specimens

Gene symbol	PVT1			CYTOR		
Relative expression level	High	Low	*P*	High	Low	*P*
**Gender, Female / Male**	22 / 27	14 / 35	0.094	14 / 33	22 / 29	0.171
**Age at diagnosis, year**	43.65±2.73	39.42±2.44	0.252	43.10±2.98	40.10±2.22	0.417
**KPS score, >80 / ≤80**	38 / 11	47 / 2	0.007	40 / 7	45 / 6	0.648
**GFAP (low / high)**	0 / 43	3 / 37	0.067	1 / 38	2 / 42	0.629
**Ki-67 (low / high)**	19 / 24	31 / 9	0.002	18 / 21	32 / 12	0.014
***MGMT* promoter methylation (- / +)**	40 / 1	39 / 1	0.986	36 / 1	43 / 1	0.901
***IDH* mutation (- / +)**	24 / 17	26 / 14	0.550	29 / 8	21 / 23	0.005
**P53 (low / high)**	11 / 30	24 / 16	0.003	10 / 27	25 / 19	0.007
**1p / 19q codeleted (- / +)**	10 / 8	10 / 10	0.732	9 / 5	11 / 13	0.272

**Table 2B:** Correlations between lncRNA (HAR1A and MIAT) expression and pathology parameters of diffuse glioma specimens

Gene symbol	HAR1A			MIAT		
Relative expression level	High	Low	*P*	High	Low	*P*
**Gender, Female / Male**	15 / 33	21 / 29	0.270	16 / 33	20 / 29	0.402
**Age at diagnosis, year**	39.63±2.48	43.37±2.69	0.311	40.84±2.74	42.23±2.46	0.706
**KPS score, >80 / ≤80**	44 / 4	41 / 9	0.158	45 / 4	40 / 9	0.136
**GFAP (low / high)**	2 / 38	1 / 42	0.514	0 / 43	3 / 37	0.067
**Ki-67 (low / high)**	30 / 10	20 / 23	0.008	33 / 10	17 / 23	0.001
***MGMT* promoter methylation (- / +)**	39 / 1	40 / 1	0.986	39 / 2	40 / 0	0.157
***IDH* mutation (- / +)**	20 / 20	30 / 11	0.032	26 / 15	24 / 16	0.752
**P53 (low / high)**	25 / 15	10 / 31	0.001	25 / 16	10 / 30	0.001
**1p / 19q codeleted (- / +)**	12 / 13	8 / 5	0.428	13 / 10	7 / 8	0.741

KPS, karnofsky performance score; +, positive; -, negative.

### Survival outcome evaluation of patients with chemotherapy and radiotherapy

**Figure 6:**
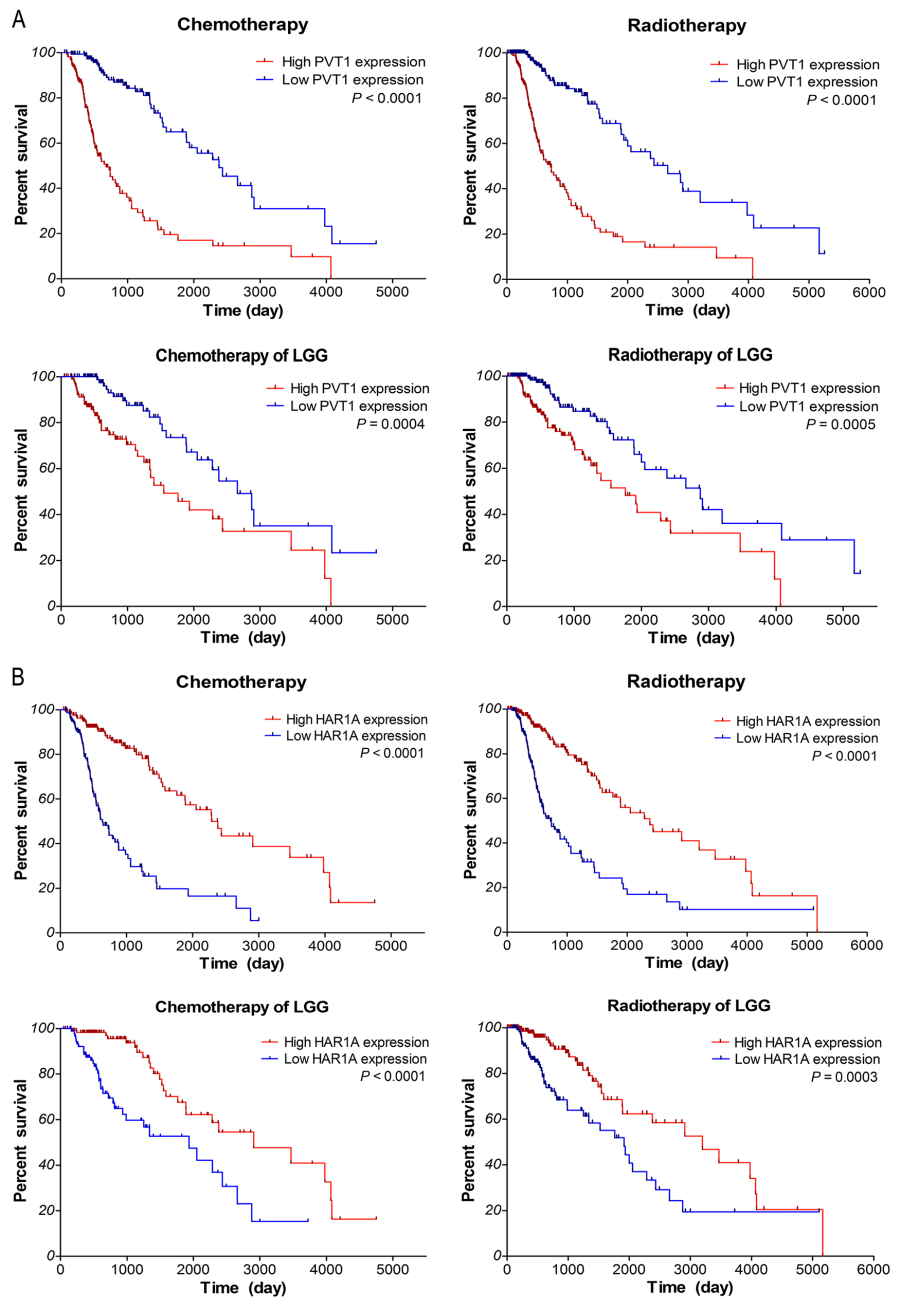
Kaplan–Meier survival analysis of patients who received chemotherapy or radiotherapy in gliomas samples of TCGA along with LGG subtypes based on the expression of PVT1 **(A)** and HAR1A **(B).** The *P*-values were computed by the log-rank test.

To determine the correlations of lncRNAs expression with the response to chemotherapy and radiotherapy, further survival analyses were performed with TCGA data, for which therapeutic information was available. It is well known that glioma patients could benefit from temozolomide (TMZ) and radiation therapy. As shown in Figure 6A-6B, within glioma samples of TCGA along with LGG subtypes, the overall survival of low PVT1 expression group was significantly better than that of high expression group in glioma patients who received chemotherapy (*P* <0.0001 and *P* =0.0004) or radiotherapy (*P* <0.0001 and *P* =0.0005). Moreover, survival of high HAR1A expression patients was noticeably better than that of low expression patients who received chemotherapy (both *P* <0.0001) or radiotherapy (*P* <0.0001 and *P* =0.0003). As for lncRNAs CYTOR and MIAT, there is no significant association of their expression with the therapeutic outcome of glioma samples (*P* >0.05; data not shown). These results suggested that glioma patients with low PVT1 expression and high HAR1A expression could benefit more from chemotherapy and radiotherapy; up-regulated PVT1 and down-regulated HAR1A might be the indicators of poor response to adjuvant chemoradiotherapy.

## DISCUSSION

Identification of potential biomarkers which can be applied for diagnosis, prognosis and as therapeutic targets has long been a priority of tumor research. Our studies first identified differentially expressed lncRNAs in glioma based on gene expression profiling, and focused on the lncRNAs PVT1, CYTOR, HAR1A and MIAT for further analysis. Results demonstrated that both PVT1 and CYTOR expressions were up-regulated, while the HAR1A and MIAT expressions were down-regulated in diffuse gliomas. High PVT1 and CYTOR expression as well as low HAR1A and MIAT expression were associated with high Ki-67 and more *TP53* mutation in diffuse glioma specimens. Meanwhile, high CYTOR expression and Low HAR1A expression were correlated with *IDH* mutation. Furthermore, survival curve and Cox regression analyses showed that glioma patients with high PVT1 expression or low HAR1A expression had poor survival outcome, aberrantly expressed PVT1 and HAR1A could be the independent prognosis biomarkers for diffuse glioma patients.

Located at the chromosome 8q24.21, human *Pvt1* oncogene encode a lncRNA PVT1, which was discovered primarily as an activator of adjacent gene *MYC* transcription [14], it has been implicated in regulating *MYC* oncogene to promote tumorigenesis [15]. Amplification of *PVT1* correlated with short survival duration of ovarian and breast cancer. Interfering PVT1 expression inhibited proliferation and induced apoptosis in breast and ovarian cancer cell lines [16]. The expression of PVT1 was increased in gastric cancer tissues compared with adjacent non-neoplastic tissues, it up-regulation was associated with lymph node metastasis and tumor-node-metastasis (TNM) stage [17]. PVT1 might serve as an independent prognostic biomarker for gastric cancer patients. Knockdown of PVT1 inhibited cell proliferation and arrested cell cycle at G1 stage via recruiting EZH2 (zeste homolog 2) and regulating p15 and p16 epigenetically [18]. Recent studies reported lncRNA PVT1 was highly expressed in pancreatic ductal adenocarcinoma (PDAC) tissues, and correlated with clinical stage. Patients with high PVT1 expression exhibited poor overall survival, PVT1 could be an independent prognostic factor for patients with PDAC [19].Salivary PVT1 were also up-regulated in the saliva of patients, might serve as potential non-invasive biomarker for diagnosis of pancreatic cancer [20]. In pancreatic cancer cells, knockdown of PVT1 inhibited cell proliferation, migration and epithelial-mesenchymal transition (EMT) by regulating p21 expression [21]. Moreover, PVT1 expression was up-regulated in colorectal cancer tissues, which correlated with the expression of MYC and many MYC regulating genes FUBP1, EZH2, and NPM1. Highly expressed PVT1 was a prognostic factor for overall survival of colorectal cancer patients. Especially, lncRNA PVT1 was detected in extracellular vesicles of colorectal cancer cells [22]. Knockdown of PVT1 inhibited cell proliferation, invasion, and activated apoptotic signaling pathway in colorectal cancer cells [8]. In hepatocellular carcinoma (HCC), hepatic oncofetal lncRNA PVT1 was up-regulated, and patients with high PVT1 expression had a poor clinical prognosis [23]. Additionally, hepatitis B virus (HBV) infection contributed to the development of early-onset HCC. HBV gene integrated into 8q24 locus may up-regulate the expression of MYC, PVT1, and miR-1204 in HCC [24]. Up-regulation of PVT1 correlated with high serum α-fetoprotein level and high recurrence rate of HCC, these indicated PVT1 could serve as a novel biomarker for predicting recurrence of HCC patients [25].

CYTOR (cytoskeleton regulator RNA), a new long intergenic non-coding RNA (also known as LINC00152), is located at chromosome 2p11.2. Previous studies have shown that expression of CYTOR was increased in gastric cancer [26], renal cell carcinoma (RCC) [27] and gallbladder cancer [28], as compared to paired non-neoplastic tissues, high CYTOR expression was positively associated with lymph node metastasis, high TNM stage, and poor over survival [9]. Up-regulated CYTOR could also be detected in plasma and exosomes from gastric cancer patients, it might act as novel blood-based biomarker for diagnosis of gastric cancer [29]. Furthermore, CYTOR contributed to cell proliferation *in vitro* and tumor growth *in vivo* via EGFR-mediated PI3K/AKT pathway [30]. CYTOR could promote gastric cancer cell cycle progression through binding to EZH2 and regulating p15 and p21 [9]. LncRNA CYTOR was up-regulated in HCC tissues, circulating CYTOR were also highly expressed in plasma samples of HCC patients, and could serve as potential biomarker for diagnosis of HHC [31]. high CYTOR expression was closely associated with HBV infection, HBx expression and poor prognosis of HCC patients. Knockdown of CYTOR inhibited HCC cell proliferation and invasion via binding to EZH2 and inhibiting E-cadherin expression [32]. Additionally, CYTOR could promote cell proliferation *in vitro* and tumor growth *in vivo* via activating the mTOR (mechanistic target of rapamycin) signaling pathway [33].

*HAR1A* (highly accelerated region 1A), a strongly evolving cis-antisense locus at the end of chromosome 20 that is specifically transcribed in human nervous system [34]. It’s consistent with our result that HAR1A was preferentially expressed in the neural subtypes of glioblastoma. Previous studies reported that HAR1A could be repressed transcriptionally by REST, and HAR1 expression was lower in the striatum of Huntington’s disease patients than normal controls [35]. In human cancers, LncRNA HAR1A had been linked to the recurrence of breast cancer [36]. MIAT (myocardial infarction associated transcript), a lncRNA has been reported to confer the risk of myocardial infarction. Aberrant of MIAT also involved in the progression of neuroendocrine prostate cancer [37] and chronic lymphocytic leukemias (CLL) [38]. Here, we first demonstrated that HAR1A and MIAT were down-regulated in diffuse gliomas. Glioma patients with low HAR1A expression had poor survival, down-regulated HAR1A, not MIAT, could be an independent prognosis biomarker for diffuse gliomas.

Finally, we evaluated the correlations between lncRNAs expression and the response to chemoradiotherapy. Survival analyses indicated that glioma patients with low PVT1 expression and high HAR1A expression could benefit more from chemotherapy and radiotherapy. Previous studies showed that PVT1 expression was increased in the tissues of cisplatin-resistant gastric and ovarian cancer patients, as well as in cisplatin-resistant cancer cells [39, 40]. Knockdown of PVT1 could reverse cisplatin-resistance in the resistant gastric cancer cells by regulating the expression of MDR1, MRP, mTOR and HIF-1α [39]. Conversely, over-expression of PVT1 contribute to the development of cisplatin resistance by regulating apoptotic pathways in ovarian cancer cells [40]. Moreover, genome-wide screening platform identified PVT1 could regulate gemcitabine sensitivity in pancreatic cancer cells ASPC1, down-regulation of PVT1 sensitize tumor cells to gemcitabine [41], these results implied that PVT1 and HAR1A may be explored as potential biomarkers for indicating therapeutic efficiency of cancer patients.

## MATERIALS AND METHODS

### Glioma gene expression datasets

The Cancer Genome Atlas (TCGA) gene expression data (Illumina HiSeq) plus clinical information for GBM and LGG (lower grade glioma) samples were obtained from TCGA data portal (http://www.cancergenome.nih.gov). The dataset included 152 GBM and 460 LGG patients, and most of them received TMZ chemotherapy and/or radiation therapy. The glioma gene expression profiling of GSE4290 [42] and GSE43378 [43] was downloaded from the Gene Expression Omnibus database (GEO, http://www.ncbi.nlm.nih.gov/geo/) [44]. In GSE4290 dataset, 23 brain tissues from epilepsy patients as non-tumor controls and 157 glioma samples (from American patients), including astrocytoma, oligodendroglioma and glioblastoma samples. Total of 50 glioma samples including grade II, III and IV from Japanese patients were analyzed in GSE43378. The original CEL files and annotation files of the platform were also downloaded. The gene expression profiling are based on Affymetrix Human Genome U133 Plus 2.0 Array platform (Affymetrix Inc., Santa Clara, CA, USA).

### Differentially expressed lncRNAs analysis

The raw data of GSE4290 was preprocessed by affy package of Bioconductor R (http://www.bioconductor.org/packages/release/bioc/html/affy.html), and probe annotation was gained via an annotation file of Affymetrix [45]. Following normalization, the differentially expressed lncRNAs (DELs) analysis between glioblastoma samples and non-tumor controls of GSE4290 dataset was performed with the Limma package of Bioconductor R (http://www.bioconductor.org/packages/release/bioc/html/limma.html). An absolute log2 fold-change (|logFC|) more than 1 and *P*-value less than 0.05 were set as cut-off point. Hierarchical clustering of 163 DELs was performed based on gene expression values of each sample to verify the difference between glioblastomas and non-tumor controls. Visualization of the identified DELs including volcano plots and heat-map was performed with the ggplot2 (http://ggplot2.org/) and gplots packages (https://cran.r-project.org/web/packages/gplots/) of R, respectively.

### Clinical specimens

Glioma tissue specimens (n=98) were obtained from patients who diagnosed with gliomas undergoing surgical resection at the Department of Neurosurgery of Xiangya Hospital of Central South University from June 2015 to October 2016. After excision, tissues were immediately frozen in liquid nitrogen for subsequent use. The clinical pathological data was assembled according to the classification of 2016 CNS WHO, and their patient information were presented in Supplementary Table 2. Twelve non-tumor tissues were obtained from adult patients with craniocerebral injuries, which required partial resections of brain tissue as decompression treatment to reduce intracranial pressure. This study was approved by the Ethics Committees of Central South University, and the patients were provided written informed consent.

### RNA extraction and qRT-PCR analysis

Total RNA was extracted from tissues using the TRIzol reagent (Invitrogen) following the manufacturer’s instructions. RNA concentration and quality were determined with UV spectrophotometer analysis at 260 nm, and RNA quality was checked by electrophoresis. One microgram total RNA of each sample was reversely transcribed into cDNA in a final volume of 20 µl under standard conditions by using PrimeScript^RT^ reagent Kit with gDNA Eraser (Takara, Japan). The synthesized cDNA was stored in −80°C for subsequent use. Quantitative real-time polymerase chain reaction (qRT-PCR) was performed with the SYBR^®^ Premix DimerEraser™ (Takara, Japan) on the LightCycler^®^ 480 system (Roche Diagnostics) according to manufacturer’s protocol. The qRT-PCR reaction included an initial denaturation step at 95°C for 30 s, followed by 40 cycles of 92°C for 5 s, 55°C for 30 s and 72°C for 30 s. ACTB (β-actin) was used as the internal control for data normalization. Relative quantification of gene expression was calculated by the comparative cycle-threshold (CT) method and presented with the values of negative ΔΔCT (−ΔΔCT).

Primers for PVT1, CYTOR, HAR1A and MIAT were designed and synthesized by Sangon Biotech (Shanghai, China). Human ACTB internal control primer (B661102-0001) was purchased from Sangon Biotech. The primer sequences were as following:

PVT1 forward primer: 5′-AGCACTCTGGACGGACTTGAGA-3′,
reverse primer: 5′- CCACTAGCAGCAACAGGAGAAG-3′;
CYTOR forward primer: 5′-ACCGAAAATCACGACTCAGCCC-3′,
reverse primer: 5′-AATGGGAAACCGACCAGACCAG-3′;
HAR1A forward primer: 5′-ACTCTGGTGTGTCCCGTTTGAAG-3′,
reverse primer: 5′-GTGCTCAAGGCTCGCTCTGTG-3′;
MIAT forward primer: 5′-GCTGACCACTAACAACCAACC-3′,
reverse primer: 5′-AGGAACAGACCAGGAAGGCAG-3′;

### Statistical analysis

The statistical analyses were performed with the SPSS 22.0 software (IBM SPSS, Chicago, USA). GraphPad Prism version 5.0 (GraphPad Software, Inc., La Jolla, USA) was used for graphing and analysis. Data were exhibited as means ± standard deviation (SD). The Pearson’s *χ*^2^ test was used to compare categorical variables. Regarding the numerical variables, statistical significance of differences between two groups was assessed using two-sided Student’s *t* test; and comparisons of multiple groups were made with one-way analysis of variance (ANOVA). *P*-value less than 0.05 was considered as a statistical difference. Survival analysis was performed via Kaplan–Meier method with the log-rank (Mantel-Haenszel) test. The risk association of lncRNA expression with several known clinical pathology factors was determined using univariate and multivariate Cox regression analyses.

## CONCLUSION

In this study, certain differentially expressed lncRNAs had been identified based on glioma gene expression profiling. Subsequent analyses demonstrated that the expressions of PVT1 and CYTOR were up-regulated, while HAR1A and MIAT were down-regulated in diffuse glioma samples compared to non-tumor tissues. Glioma patients with high PVT1 expression and low HAR1A expression had poor survival outcome, up-regulated PVT1 and down-regulated HAR1A could be the independent prognosis factors for glioma patients. Moreover, down-regulation of PVT1 and up-regulation of HAR1A might contribute to improve the survival of patients who received chemotherapy or radiotherapy. These results implied that these four lncRNAs might play important role in diffuse gliomas progression, particularly, PVT1 and HAR1A could be explored as promising biomarkers for diagnosis, prognosis and target therapy of diffuse gliomas.

## SUPPLEMENTARY MATERIALS FIGURE AND TABLES




