# Supplementary material for: lncRNAs PVT1 and HAR1A are prognosis biomarkers and indicate therapy outcome for diffuse glioma patients
Source: Oncotarget. 2017 Aug 12;8(45):78767–80. doi: 10.18632/oncotarget.20226 (PMC5667997; doi:10.18632/oncotarget.20226)
Supplement: Supplementary file 1 [file oncotarget-08-78767-s001.pdf]

lncRNAs PVT1 and HAR1A are prognosis biomarkers and indicate therapy outcome for diffuse glioma patients

SUPPLEMENTARY MATERIALS

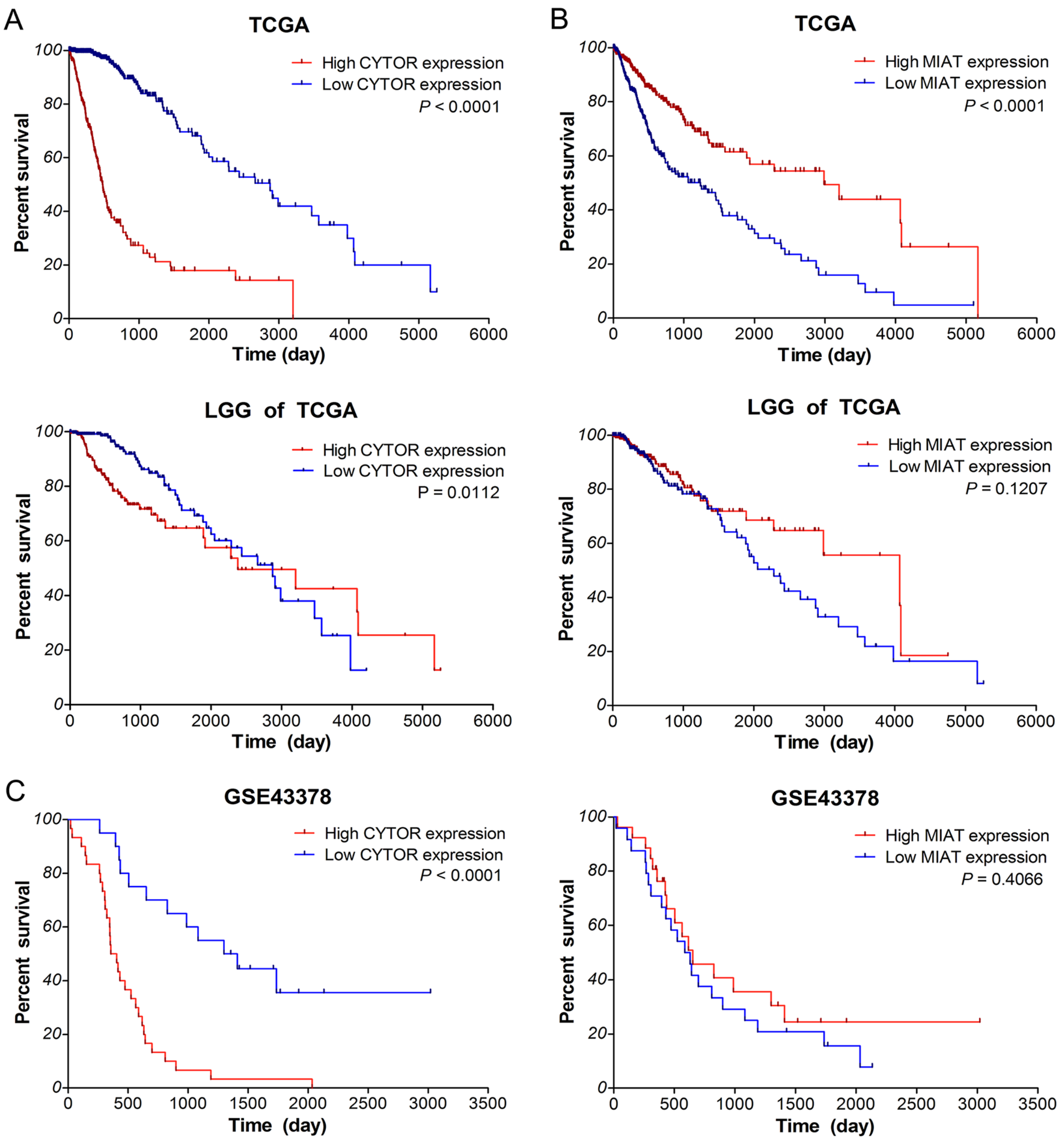

Supplementary Figure 1: Kaplan–Meier survival curve analyses with a log-rank comparison were performed based on *CYTOR* **(A)** and *MIAT* **(B)** expressions in gliomas samples of TCGA along with LGG subtypes, and gliomas samples of GSE43378 **(C)** datasets.

Supplementary Table 1: Probe sets of lncRNAs in the annotation file of Affymetrix HG-U133 Plus 2.0 arrays

See Supplementary File 1

Supplementary Table 2: Clinical and molecular pathology characteristics of diffuse glioma patients

Clinical characteristics	Specimens
Number of patients (n)	98
Oligodendroglioma	13
Oligoastrocytoma	10
Astrocytoma	49
Glioblastoma with grade IV	26
WHO grade II	40
WHO grade III	32
Gender, female / male	36/62
Age at diagnosis, year	45.34±1.543
KPS score, >80 / ≤80	85/13
GFAP (low / high)	3/80
Ki-67 (low / high)	50/33
MGMT promoter methylation (- / +)	79/2
IDH mutation (- / +)	50/31
P53 (low / high)	35/46
1p/19q codeleted (- / +)	20/18

KPS, karnofsky performance score; +, positive; -, negative.
